# 

**Published:** 1898-02-26

**Authors:** 


**Notices of Births, Deaths, and Marriages** are inserted in this column at a charge of 2s. 6d. for an announcement not exceeding four lines.

### NOTICE TO CORRESPONDENTS.

All MS., Letters, Books for Review, and other matters intended for the Editor should be addressed The Editor, "The Hospital," The Hospital Building, 28 & 29, Southampton Street, Strand, W.C.

The Editor cannot undertake to return rejected MS., even when accompanied by stamped directed envelope.

**Notice to Advertisers and Subscribers.**

All Advertisements, Orders for Copies of The Hospital, and Business Communications should be addressed to **THE MANAGER, (Not to The Editor),** The Hospital, The Hospital Building, 28 & 29, Southampton Street, Strand, London, W.C.

The Cost of Subscription, post free, is as follows:—Per week, 2½d. per quarter, 2s. 8d.; per half-year, 5s. 3d.; per annum, 10s. 6d. Foreign:—Per annum, 15s. 2d.; payable, in all cases, in advance.

**NOTICE.—Vol. XXII. of "The Hospital" (April, 1897, to September, 1897), in handsome cloth binding, is now on sale at the Office of this Journal, price 7s. 6d. Cases for binding the Numbers contained in this Volume, 1s. 6d. Index to Vol. XXII., 2½d., post free.**

**The Monthly Part for March will be ready on March 1st. Price 6d., by post 8d.**

### SCRAPS AND GLEANINGS.

THE medical report of the Bradford Children's Hospital shows that 460 children were admitted to the hospital during the year, and that the out-patients made 7,418 attendances.

THE number of in-patients treated at the West Cornwall Infirmary at Penzance in 1897 was 145, a slight decrease on the previous year. The number of out-patient tickets entered was 1,819.

INCLUDING those remaining at the close of last year, 1,420 patients were treated in the wards of the Glasgow Eye Infirmary in 1897. The number of new cases at the dispensaries was 17,667.

THE number of patients treated at the West Kent General Hospital shows an all round increase. In-patients numbered 426 in 1897, against 398 in 1896; out-patients 812 and 794; and casualties 1,085 and 943 respectively.

DURING 1897, 92 in and 2,503 out patients were treated at the Tunbridge Wells Homœopathic Hospital. In addition, 2,203 visits were made to home patients.

THE annual general meeting of the Society for the Protection of Birds will take place on Friday, February 25th, 1898, at the Westminster Palace Hotel, Victoria Street, S.W.

Two thousand four hundred and twenty-eight new provident members were added to the roll of the Reading Dispensary last year, making a total of 18,551 at the present time.

FORRES LEANCHOIL HOSPITAL shows an increase of one in the number of patients treated in 1897 (78) when compared with 1896.

ONE hundred and forty in and 705 out patients were attended at the Sussex Eye Hospital, Brighton, during the past year.

DURING the past ten years the work at the Leeds Hospital for Women and Children has increased about 50 per cent. In-patients numbered 391 in 1897, and out-patients 2,832.

IN the forty-sixth year of its existence (1897) the Liverpool Infirmary for Children treated 1,196 in and 14,211 out patients.

THE Liverpool Dental Hospital had 24,258 patients in 1897.

THE committee of the Tiverton Infirmary state that the past year has been the worst financially for the institution since its foundation. One thousand seven hundred and ninety-eight cases were treated.

THE new workhouse Infirmary for Newton has been formally opened.

THE Accrington Medical Dispensary, which was started in July last by the members of the old dispensary, now numbers 1,032 members.

THE plans for the new building of the Gainsborough Cottage Hospital have been approved.

THE foundation stone of the Croydon Rural District Council's Isolation Hospital at Beddington Corner was laid last month.

THE plans for the new workhouse infirmary at Plymouth have been approved.

ANNUAL subscriptions in 1896-7 to the Northern Counties Hospital for Incurables at Mauldeth show a gratifying increase. The system of out-pensioners inaugurated in May last has proved a success.

DURING the year 1896-97 616 patients were treated at the Guest Hospital, Dudley. In consequence of the extensions which were opened on Jubilee Day, the Committee estimate that at least a further £300 a-year will be required to meet the increased liabilities which the hospital must incur.

THE report of the Committee of the Manchester Hospital Sunday and Saturday Fund states that early in the past year representations were made by the Red Cross Society (the working-men's auxiliary of the Fund) that the workpeople objected to the charges made to patients at some of the Manchester hospitals, and would not adopt the Society's system of collections unless some modification of the system of collections from patients were made. The Board of the Manchester Royal Infirmary, on being approached, did not see its way to make any concession in favour of any section of contributors to the funds of that hospital. The amount of collections were £8,200 in 1895, £8,315 in 1896, and £8,088 in 1897.

### The Student of Medicine.

### PAPERS SET AT THE EXAMINATION OF CANDIDATES FOR HER MAJESTY'S ARMY AND INDIAN MEDICAL SERVICES.

### SURGERY.

Sir William MacCormac, Bart.—February 4th, 1898.

(*All four questions to be answered.*)

1. Enumerate the complications which may occur in a case of otitis media. Give the symptoms and treatment of abscess of the temporo-sphenoidal lobe dependent upon this cause.
2. Write an account of the course of a case of typical syphilis. Give the treatment you would adopt, and mention any matters of importance in arriving at a prognosis.
3. Mention two causes which produce ulceration of the cornea. Give a brief account of the treatment you would adopt in each case, the possible complications which may result, and the manner of dealing with them.
4. Give a description, pathological as well as clinical, of a case of carcinoma of the breast. Describe the varieties of the disease met with, and, in the event of an operation being performed, mention any operative details you consider important.

### CHEMISTRY AND MATERIA MEDICA.

Dr. Norman Moore.—February 4th, 1898.

1. Describe an experiment demonstrating the chemical composition of water.
2. What are the properties of phosphorus? Describe its modifications, and mention (with their formulæ) the acids into the composition of which it enters.
3. What are the preparations (in the Pharmacopeia) of each of the following metals: mercury, bismuth, iron?
4. Mention the chief expectorants of the Pharmacopeia. State the dose of each (*a*) for an adult, (*b*) for a child of five years of age.
5. Name the chief drugs used to procure sleep, and the dose of each. What precautions ought to be observed in their administration?

### MEDICINE AND PATHOLOGY.

Professor McCall Anderson.—February 5th, 1898.

1. What is your opinion with regard to the following case; Give the grounds for your diagnosis; say what treatment you would recommend; and, in the event of a fatal issue, what would you find post mortem.

A soldier, aet. 32, was admitted into hospital on December 4th, 1897. He stated that he had always enjoyed fair health, but admitted that he had led a very irregular life. He complained of debility, some loss of flesh, excessive urination, and, above all, of a right internal squint of some weeks' duration; indeed, it was this which induced him to seek advice.

On examination he was found to be pallid, rather weakly, and slightly emaciated, but there was no fever. The internal squint was pronounced, and he was quite unable to turn the eye outwards. There were no head symptoms, and the heart and lungs were healthy, while the digestion was fair. But the liver was greatly and uniformly enlarged; it felt very firm, and was not the seat of either pain or tenderness. The urine was very pale, 120 ounces per day, specific gravity, 1013; it contained a small quantity of albumen, and an occasional granular or hyaline tube cast was discovered in the scanty deposit.

Finally, on one shoulder there was a patch of eruption, about the size of the hand, which had appeared about eight months before admission. It was composed of tubercles of a dusky red tint, with here and there an admixture of violet. Some of these had ulcerated and were capped with greenish crusts. The edge of the patch was rounded, abrupt, and elevated.

2. About what day of the fever does the eruption make its appearance in the following diseases; (*a*) Rubella (German measles), (*b*) morbilli (measles), (*c*) scarlatina, (*d*) typhus, (*e*) enteric fever, (*f*) varicella, (*g*) variola?
3. How would you treat typical uncomplicated cases of (*a*) lead colic, (*b*) ulcer of the stomach (*c*) acute tubular nephritis?
4. Write down what you know with regard to Beriberi.

### ANATOMY AND PHYSIOLOGY.

Mr. Makins.—February 5th, 1898.

1. Describe the urinary bladder in the male, and give its relations to surrounding structures.
2. Give a short account of the anatomy and functions of the tympanum and its annexes.
3. Trace the course and distribution of the musculo spinal nerve from its origin to a point on a level with the external condyle of the humerus.
4 Describe the sequence of changes which carbo-hydrates and fats undergo in their passage through the body.

### NATURAL SCIENCES.

Dr. Norman Moore.—February 11th, 1898.

(Candidates may answer not more than *six* questions, and they must confine themselves to *two* branches of science only).

*Zoology and Comparative Anatomy.*

1. Describe the arrangement of the bones in the fore-limb of a horse. Point out how the forelimb of a horse differs from and how it resembles the forelimb of a man.
2. Describe the heart and circulatory system of an osseous fish.
3. Give an account of the structure and life history of amœba.

*Botany.*

1. A tree weighing 12 pounds when planted in a pot of earth in 1894 is found in 1898 to have doubled in weight. The pot is unchanged in weight. The earth weighs 4 ounces less than in 1894.

Explain in what way the tree has obtained its increased weight of 12 pounds.

2. Give the characteristics of the following natural orders: Malvaceæ, rosaceæ, labiatæ, scrophulariaceæ, solanaceæ.

3. Describe the method of fertilization in wheat, in the date palm, and in any species of orchis with which you may be acquainted.

*Physics.*

1. What is meant by the specific gravity of a substance?

How would you ascertain the specific gravity of a diamond?

2. Describe experiments illustrating conduction of heat and convection of heat.
3. Describe the structure and use of the gold-leaf electroscope.

*Physical Geography and Geology.*

1. In what strata do the remains of the following animals occur, and in which they are most abundant: Encrinites, ammonites, large saurians, marsupial mammals?
2. Describe the appearances which would indicate that a particular tract of land had once been occupied by a fresh water lake.
3. Define the following terms: (1) Fault. (2) Isothermal lines. (3) Metamorphic rocks. (4) Granite.

